# Multi-institutional development and external validation of machine learning-based models to predict relapse risk of pancreatic ductal adenocarcinoma after radical resection

**DOI:** 10.1186/s12967-021-02955-7

**Published:** 2021-06-30

**Authors:** Xiawei Li, Litao Yang, Zheping Yuan, Jianyao Lou, Yiqun Fan, Aiguang Shi, Junjie Huang, Mingchen Zhao, Yulian Wu

**Affiliations:** 1 Department of Surgery, Second Affiliated Hospital, Zhejiang University School of Medicine, Hangzhou, 310000 Zhejiang China; 2 Key Laboratory of Cancer Prevention and Intervention, China National Ministry of Education, Cancer Institute, Second Affiliated Hospital, Zhejiang University School of Medicine, Hangzhou, 310000 Zhejiang China; 3 Cancer Center, Zhejiang University, Hangzhou, 310058 Zhejiang China; 4 Department of Surgery, Cancer Hospital of the University of Chinese Academy of Sciences (Zhejiang Cancer Hospital), Hangzhou, 310000 Zhejiang China; 5 Hessian Health Technology Co., Ltd, Beijing, 100007 China; 6 Department of Surgery, Fourth Affiliated Hospital, Zhejiang University School of Medicine, Yiwu, 322000 Zhejiang China; 7 Department of Surgery, Changxing People’s Hospital, Huzhou, 313100 Zhejiang China

**Keywords:** Machine learning, PDAC, Relapse, Prediction model, Radical surgery

## Abstract

**Background:**

Surgical resection is the only potentially curative treatment for pancreatic ductal adenocarcinoma (PDAC) and the survival of patients after radical resection is closely related to relapse. We aimed to develop models to predict the risk of relapse using machine learning methods based on multiple clinical parameters.

**Methods:**

Data were collected and analysed of 262 PDAC patients who underwent radical resection at 3 institutions between 2013 and 2017, with 183 from one institution as a training set, 79 from the other 2 institution as a validation set. We developed and compared several predictive models to predict 1- and 2-year relapse risk using machine learning approaches.

**Results:**

Machine learning techniques were superior to conventional regression-based analyses in predicting risk of relapse of PDAC after radical resection. Among them, the random forest (RF) outperformed other methods in the training set. The highest accuracy and area under the receiver operating characteristic curve (AUROC) for predicting 1-year relapse risk with RF were 78.4% and 0.834, respectively, and for 2-year relapse risk were 95.1% and 0.998. However, the support vector machine (SVM) model showed better performance than the others for predicting 1-year relapse risk in the validation set. And the k neighbor algorithm (KNN) model achieved the highest accuracy and AUROC for predicting 2-year relapse risk.

**Conclusions:**

By machine learning, this study has developed and validated comprehensive models integrating clinicopathological characteristics to predict the relapse risk of PDAC after radical resection which will guide the development of personalized surveillance programs after surgery.

**Supplementary Information:**

The online version contains supplementary material available at 10.1186/s12967-021-02955-7.

## Introduction

Pancreatic ductal adenocarcinoma (PDAC) is one of the most lethal human malignant diseases worldwide and the sixth leading cause of cancer-related deaths in China [1]. So far, radical resection followed by adjuvant chemotherapy has been the only potentially curative treatment [2]. However, only a minority of patients present with a tumor suitable for this combination therapy at diagnosis, due to lack of early clinical symptoms and effective screening approaches [3]. Even after curative resection, up to 80% of patients will suffer from disease relapse resulting in a 5-year survival of only 20–30% [4–7]. Hence, the survival of patients with resectable PDAC is closely related to recurrence. It is necessary and urgent to build robust models to identify those patients with increased risk of relapse and further optimize treatment decision-making.

Nowadays, development of methods to predict treatment outcomes and prognosis is an important paradigm in the realm of personalized medicine [8]. Several studies have shown comparable prediction accuracy by using traditional regression-based statistical methods on a basis of a combination of biomarkers and multiple clinical factors [9–12]. However, common statistical methods familiar to clinicians ignore more complex non-linear interactions between variables that might play significant roles in the potential of future relapse, and which could be captured using more sophisticated modeling approaches [13]. In recent years, machine learning, as a branch of artificial intelligence (AI) technology, has attracted extensive interest in developing clinical predictive tools for diagnosis, staging and prognosis of various diseases [14–16]. It has been successfully applied for recognizing hidden patterns in complex data, allowing for better predictions of clinical outcomes than conventional statistical models, especially when applied to large-scale datasets [17].

Thus, the aim of this study was to develop, and externally validate, new cutting-edge machine learning-based models that accurately predict 1- and 2-year relapse of PDAC using clinicopathological factors in patients with resectable disease. Predicting the risk of relapse offers the potential to improve personalized surveillance schedules, determine clinical trial eligibility and compare results across studies and different institutions [18].

## Materials and methods

### Study population

Data of PDAC patients who underwent radical resection at 3 institutions between January 2013 and December 2017 were obtained. The study was approved by the Institutional Review Boards of 3 institutions. And no additional patient consent was required since the medical records were retrospectively reviewed. As this study aimed to build models based on preoperative clinical and pathological factors affecting relapse risk after surgery in resectable PDAC, patients who had initially borderline resectable/unresectable cancers according to the NCCN guideline [19] or received neoadjuvant therapy were excluded. So were those who were lost to follow-up or lacking complete clinical data. The inclusion criteria were met by a total of 262 patients, including 183 from the Second Affiliated Hospital of Zhejiang University School of Medicine, 70 from the Cancer Hospital of the University of Chinese Academy of Sciences and 9 from the Fourth Affiliated Hospital of Zhejiang University School of Medicine.

### Data collection

Preoperative blood biomarkers including carcinoembryonic antigen (CEA), CA199, CA125, white blood cell (WBC) count, hemoglobin (Hb) count, platelet (Plt) count, neutrophil (Neut) count, lymphocyte (Lymp) count, monocyte (Mono) count, albumin (Alb), globulin (Glb), aspartate transaminase (AST), alanine transaminase (ALT), alkaline phosphatase (ALP), gamma-glutamyltransferase (GGT), total bilirubin (TB) and direct bilirubin (DB) were collected using the measurements that were closest to the operation and within at least 1 week before the surgery. Inflammation-based prognostic scores, including albumin-globulin ratio (AGR) [20], lymphcyte-monocyte ratio (LMR) [21], neutrophil–lymphocyte ratio (NLR) [22] and platelet-lymphocyte ratio (PLR) [23], were calculated. Additionally, pathological diagnosis and description was carried out by experienced pancreatic pathologists at 3 institutions, including surgical margin status, tumor site, tumor size, tumor differentiation, T-stage, lymph node status (N-stage), vascular invasion, perineural invasion and adipose tissue invasion.

After surgery, the follow-up of patients was initially performed every 3 months for the first 2 years, every 6 months during years 3 and 4, and then annually. The surveillance protocol included physical examination, serum CA19-9 level and contrast-enhanced abdominoperineal computed tomography (CT). When imaging features were consistent with a cancer recurrence, magnetic resonance imaging (MRI) and/or fluorodeoxyglucose positron emission tomography (PET) was carried out to further clarify ambiguous CT findings if necessary. Relapse-free survival (RFS) and overall survival (OS) were defined as the duration from the date of surgery until the date when a relapse was diagnosed and death, respectively, or last follow-up.

### Statistical analysis

Differences of clinical characteristics between the training set and the validation set as well as between patient groups with or without 1- and 2-year relapse were assessed using independent sample t test, Mann–Whitney U test, or χ^2^ test with a statistical significance level set at 0.05. Clinical variables found significantly different (*p* < 0.05) between patient groups with or without 1- and 2-year relapse were selected as inputs for the predictive models.

In our study, six algorithms were applied to build models for predicting 1- and 2-year relapse. In addition to the basic binary LR model, several machine learning models were developed: random forest (RF), support vector machine (SVM), gradient boosting machine (GBM), Neural network (NN), k neighbor algorithm (KNN). RF and GBM both are tree-based ensemble algorithms. RF creates multiple decision tree models by bootstrap samples, and aggregates decisions through averaging or majority voting [24]. And GBM uses all the data to build a regression tree model from the beginning, and constructs the new models to be maximally correlated with the negative gradient of the loss function [25]. SVM provides two-class prediction by constructing the separating hyperplane that has the largest distance to the nearest training data points from each of the two classes [26]. The neural network algorithm recognizes the potential relationships in a set of data through constructing a network structure composed of three main layers (input, hidden and output layer) and the main task is to transform raw input units into useful output units [27]. The K-nearest neighbor algorithm is based on analogical reasoning, it stores all the training data and classifies the new data point based on similarity measures [28].

For data standardizing, we centered and scaled the input features to the same range of values with mean of zero prior to modeling. Model tuning were carried out using the repeated fivefold cross-validation method with the training set. Repeated cross-validation means repeating the procedure of cross-validation for k times (k = 3 in this study), each time with different splits. The model assessment metric was calculated in each repetition and finally averaged as the final result. Compared with performing cross-validation only once, repeated cross-validation can improve estimated performance of a chosen model [29]. In each cross-validation, we tried all possible combinations of parameters by grid search. For each set of parameters, we used 4/5 of the data to fit the model, and the remaining 1/5 was assessed to compute the performance measure. Here we selected accuracy as the performance measure, which was calculated 5 times and averaged to produce the performance score of each parameter set. The ranges of training parameters for grid search were provided in Additional file 1: Table S1. Relative variable importance was calculated and plotted to find out the impact of features on the predictive models.

The performance of the final models was assessed in the validation set. The evaluation indicators used to compare the performance of models were AUROC, sensitivity, specificity, accuracy, positive predictive value (PPV), negative predictive value (NPV), F1 score and root mean squared error (RMSE). To further evaluate the performance of the models, we used bootstrapping resampling (2000 times) to compute the 95% confidence interval (CI) of AUROC and compared the AUROCs of machine learning models using 2-sided test. Finally, 95%CI of AUROCs and *p* values from comparisons were plotted together. We determined the best machine learning models for prediction of 1- and 2-year relapse with the validation set. Calibration curves were constructed to regress observed data against model fits of the best machine learning models. We also tried other variable sets as inputs for these ML models: (1) all the 32 clinical variables, (2) variables obtained through fivefold cross-validation Lasso analysis.

All statistical analysis was performed with R 4.0.2. The R package ‘caret’ was used for data pre-processing, model training (SVM and KNN), and calculation of variable importance. The R packages ‘randomForest’, ‘gbm’ and ‘nnet’ were used for the RF, GBM, and NNET model training, respectively. Lasso analysis was performed by the R package ‘glmnet’.

## Results

### Basic characteristics

The clinicopathological characteristics of the training set and validation set are shown in Table 1. 183 from the Second Affiliated Hospital of Zhejiang University School of Medicine were included as the training set. 70 from the Cancer Hospital of the University of Chinese Academy of Sciences and 9 from the Fourth Affiliated Hospital of Zhejiang University School of Medicine were used as the external independent validation cohort. Several clinical features were found significantly different between the training and validation datasets including globulin (Glb), albumin-globulin ratio (AGR), tumor differentiation, T-stage, lymph node status (N-stage) and vascular invasion (VI).

**Table 1:** Characteristics of the study population in training set and validation set

Variables		Training (n = 183)	Validation (n = 79)	*P*
Age (years)	Median (q1–q3)	63.0 (56.0–70.0)	63.0 (59.0–67.5)	0.881
Gender	Male (%)	115 (62.8)	43 (54.4)	0.255
	Female (%)	68 (37.2)	36 (45.6)	
BMI (kg/m^2^)	Median (q1–q3)	22.4 (20.3–23.9)	21.8 (19.9–24.1)	0.353
CEA (ng/mL)	< 5 (%)	140 (76.5)	51 (64.6)	0.065
	≥ 5 (%)	43 (23.5)	28 (35.4)	
CA199 (U/mL)	< 37 (%)	49 (26.8)	16 (20.3)	0.334
	≥ 37 (%)	134 (73.2)	63 (79.7)	
CA125 (U/mL)	< 35 (%)	147 (80.3)	68 (86.1)	0.349
	≥ 35 (%)	36 (19.7)	11 (13.9)	
WBC (*10^9)	Median (q1–q3)	6.0 (4.8–7.3)	5.7 (4.4–6.8)	0.220
Hb (g/L)	Median (q1–q3)	127.0 (116.0–140.0)	129.0 (120.0–142.0)	0.110
Plt (*10^9)	Median (q1–q3)	191.0 (154.5–232.0)	204.0 (164.0–265.5)	0.132
Neut (*10^9)	Median (q1–q3)	3.9 (2.9–4.8)	3.4 (2.4–4.6)	0.093
Lymp (*10^9)	Median (q1–q3)	1.4 (1.1–1.7)	1.5 (1.1–1.9)	0.275
Mono (*10^9)	Median (q1–q3)	0.5 (0.4–0.6)	0.4 (0.3–0.6)	0.295
Alb (*10^9)	Median (q1–q3)	40.4 (37.3–43.3)	40.4 (36.4–43.4)	0.622
Glb (*10^9)	Median (q1–q3)	26.7 (24.3–29.5)	28.2 (26.1–31.9)	0.002
AGR	Median (q1–q3)	1.5 (1.4–1.7)	1.4 (1.2–1.6)	0.005
NLR	Median (q1–q3)	2.7 (2.0–4.2)	2.4 (1.6–3.3)	0.153
LMR	Median (q1–q3)	3.0 (2.1–4.2)	3.5 (2.0–5.1)	0.113
PLR	Median (q1–q3)	137.1 (106.3–184.1)	140.0 (96.6–217.2)	0.528
AST (U/L)	Median (q1–q3)	44.0 (20.5–111.5)	31 (20–90.5)	0.448
ALT (U/L)	Median (q1–q3)	50.0 (17.0–200.5)	29.0 (17.0–139.0)	0.119
ALP (U/L)	Median (q1–q3)	156.0 (89.5–391.5)	105.0 (67.5–367.0)	0.257
GGT (U/L)	Median (q1–q3)	159.0 (25.0–704.0)	51.0 (20.0–494.0)	0.162
TB (μmol/L)	Median (q1–q3)	22.1 (12.0–177.3)	14.6 (9.4–134.1)	0.716
DB (μmol/L)	Median (q1–q3)	6.4 (2.6–105.2)	5.5 (3.2–109.2)	0.360
Location	Head-isthmus (%)	139 (76.0)	54 (68.4)	0.259
	Body-tail (%)	44 (24.0)	25 (31.6)	
Margin	R0 (%)	176 (96.2)	79 (100.0)	0.106
	R1 (%)	7 (3.8)	0 (0.0)	
T stage	1 (%)	43 (23.5)	2 (2.5)	< 0.001
	2 (%)	86 (47.0)	55 (69.6)	
	3 (%)	54 (29.5)	22 (27.8)	
N stage	0 (%)	105 (57.4)	36 (45.6)	0.009
	1 (%)	56 (30.6)	39 (49.4)	
	2 (%)	22 (12.0)	4 (5.1)	
VI	Yes (%)	83 (45.4)	20 (25.3)	0.004
	No (%)	100 (54.6)	59 (74.7)	
PI	Yes (%)	143 (78.1)	67 (84.8)	0.283
	No (%)	40 (21.9)	12 (15.2)	
ATI	Yes (%)	82 (44.8)	43 (54.4)	0.195
	No (%)	101 (55.2)	36 (45.6)	
Differentiation	Well (%)	37 (20.2)	5 (6.3)	0.019
	Moderate (%)	133 (72.7)	68 (86.1)	
	Poor or undifferentiated (%)	13 (7.1)	6 (7.6)	
OS	Median (q1–q3)	19.0 (11.0–33.0)	14.0 (9.0–28.0)	0.340
RFS	Median (q1–q3)	11.0 (6.0–22.8)	8.0 (4.0–19.5)	0.503
1-year relapse	Yes (%)	106 (57.9)	49 (62.0)	0.629
	No (%)	77 (42.1)	30 (38.0)	
2-year relapse	Yes (%)	138 (75.4)	59 (74.7)	1.000
	No (%)	45 (24.6)	20 (25.3)	

*BMI* body mass index, *CEA* carcinoembryonic antigen, *CA* cancer antigen, *WBC* white blood cell, *Hb* Hemoglobin, *Plt* Platelet, *Neut* neutrophil, *Lymph* lymphocyte, *Mono* monocyte, *Alb* albumin, *Glb* globulin, *AGR* albumin-globulin ratio, *NLR* neutrophil–lymphocyte ratio, *LMR* lymphcyte-monocyte ratio, *PLR* platelet-lymphocyte ratio, *AST* aspartate transaminase, *ALT* alanine transaminase, *ALP* alkaline phosphatase, *GGT* gamma-glutamyltransferase, *TB* total bilirubin, *DB* direct bilirubin, *VI* vascular invasion, *PI* perineural invasion, *ATI* adipose tissue invasion, *OS* overall survival, *RFS* relapse-free survival

Comparison of characteristics between patients with and without 1- or 2-year relapse in training set was shown in Additional file 1: Table S2 and S3, respectively. According to the univariate analysis, significant differences were observed in various clinical parameters (CA199, N stage, vascular invasion, adipose tissue invasion, differentiation) for 1-year relapse, and (CA199, N stage, vascular invasion, monocyte counts, albumin, AGR) for 2-year relapse. These variables were then included in the construction of machine learning models to predict the relapse risk of PDAC after radical surgery.

### Model performance

**Fig. 1:**
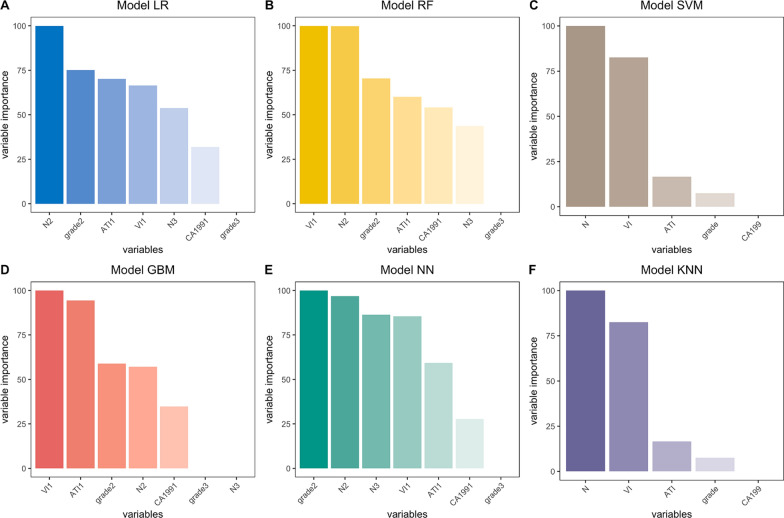
Relative importance of variables on models to predict 1-year relapse. Interpretation: N2 = N stage 1, N3 = N stage 2; grade 2 = moderate differentiation, grade 3 = poor differentiation or undifferentiated; ATI1 = with adipose tissue invasion; VI1 = with vascular invasion; CA1991 = CA 199 ≥ 37U/mL

**Fig. 2:**
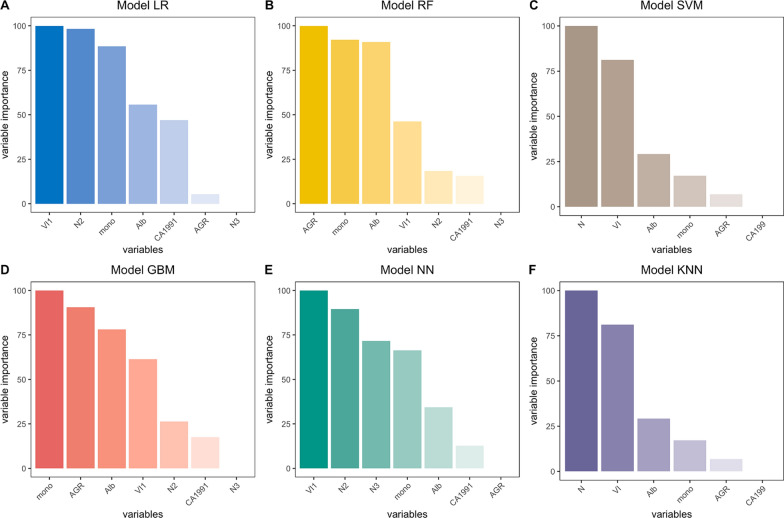
Relative importance of variables on models to predict 2-year relapse. Interpretation: VI1 = with vascular invasion; N2 = N stage 1, N3 = N stage 2; Mono = monocyte; Alb = Albumin; AGR = albumin-globulin ratio; CA1991 = CA 199 ≥ 37U/mL

Six models including LR, RF, SVM, GBM, KNN and NN were built and externally validated and the optimal parameters of these models were shown in Additional file 1: Table S4. Relative importance of variables was calculated and shown in Figs. 1 and 2. Pathological characteristics such as lymph node status (N-stage), tumor differentiation, and vascular invasion were found to have a major impact on most predictive models.

**Fig. 3:**
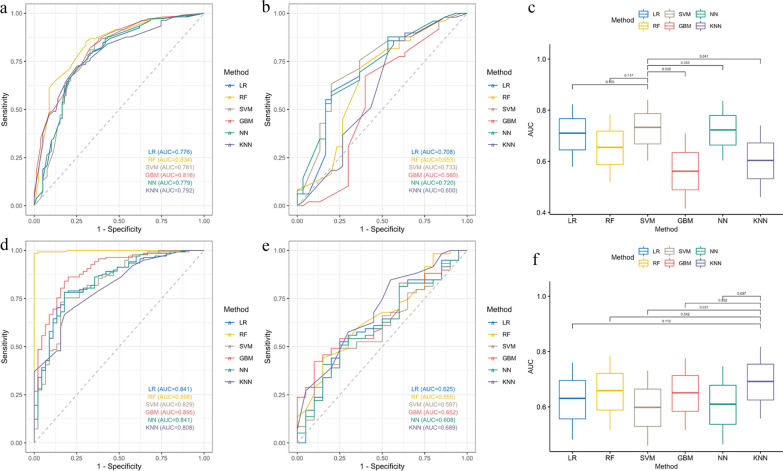
Comparisons of ROC curves and AUROC of different models to predict 1- and 2-year relapse in training cohort and validation sets (1-year relapse: training set: **A**, validation set: **B**, comparison of AUROC in validation set: **C**; 2-year relapse: training set: **D**, validation set: **E**, comparison of AUROC in validation set: **F**)

Comparisons of ROC curves and AUROC of different models to predict 1- and 2-year relapse in training cohort and validation sets were shown in Fig. 3 and Additional file 1: Figure S1. All six methods had excellent performance in the training set. Among them, the RF model outperformed the others in the training set. The highest accuracy and AUROC for predicting 1-year relapse risk with RF were 78.4% and 0.834, respectively; and for 2-year relapse risk were 95.1% and 0.998, respectively. LR obtained the lowest AUROC value of 0.776 to predict 1-year relapse risk and KNN of 0.808 to predict 2-year relapse risk.

In the validation set, the SVM model showed better performance than the others for predicting 1-year relapse risk with an accuracy and AUROC of 70.9% and 0.733, respectively (Table 2). And the KNN model achieved the highest accuracy and AUROC for predicting 2-year relapse risk of 73.4% and 0.689, respectively (Table 3). We further separately compared these two models with the rest using the AUROC. However, there was no significant statistical difference between RF and either of these two models, implying that these models might be similar in terms of their predictive power.

**Table 2:** Performance comparison of different models to predict 1-year relapse in the validation set

Model	AUC	95%CI.lower	95%CI.upper	Sensitivity	Specificity	Accuracy	PPV	NPV	F1	RMSE
LR	0.708	0.579	0.823	0.878	0.400	0.696	0.705	0.667	0.782	0.448
RF	0.653	0.519	0.782	0.837	0.400	0.671	0.695	0.600	0.759	0.501
SVM	0.733	0.603	0.840	0.857	0.467	0.709	0.724	0.667	0.785	0.445
GBM	0.560	0.416	0.708	0.776	0.367	0.620	0.667	0.500	0.717	0.509
NN	0.720	0.604	0.836	0.878	0.400	0.696	0.705	0.667	0.782	0.448
KNN	0.600	0.460	0.740	0.837	0.467	0.696	0.719	0.636	0.774	0.496

**Table 3:** Performance comparison of different models to predict 2-year relapse in the validation set

Model	AUC	95%CI.lower	95%CI.upper	Sensitivity	Specificity	Accuracy	PPV	NPV	F1	RMSE
LR	0.625	0.482	0.760	0.847	0.350	0.722	0.794	0.438	0.820	0.467
RF	0.655	0.518	0.784	0.898	0.250	0.734	0.779	0.455	0.835	0.431
SVM	0.597	0.460	0.731	0.831	0.200	0.671	0.754	0.286	0.790	0.471
GBM	0.652	0.517	0.776	0.831	0.250	0.684	0.766	0.333	0.797	0.463
NN	0.608	0.464	0.747	0.831	0.200	0.671	0.754	0.286	0.790	0.450
KNN	0.689	0.558	0.817	0.915	0.200	0.734	0.771	0.444	0.837	0.416

In addition, we also built models based on all the 32 clinical variables or variables obtained from fivefold cross-validation Lasso analysis. Nonetheless, no better predictive performance was achieved by either of these two approaches (Additional file 1: Tables S5 and S6). We still used the results from univariate analysis considering its simplicity and good performance.

Finally, we used a calibration curve to assess the agreement between the predicted and observed risks of relapse of PDAC. Adequate consistency was displayed in the training set between estimated risks using the predictive models and the actual observed outcome. However, SVM and KNN showed relatively poorer calibration performance in the validation set due to a smaller sample size (Additional file 1: Figure S2).

## Discussion

The development of predictive tools for individual relapse risk assessment after multimodal therapy may help to further optimize treatment decision-making [30]. In this study, we have constructed and validated comprehensive models integrating clinicopathological characteristics to predict the relapse risk of PDAC after radical resection. It turned out that machine learning techniques were superior to conventional regression-based analyses in terms of the predictive performance. In accordance with various studies investigating the prognostic factors of PDAC [11, 31, 32], lymph node status (N-stage), vascular invasion and CA199 are independent predictors for both 1- and 2-year relapse. Although the RF model had the highest AUROC in the training set, the SVM model and KNN model showed better robustness to predict 1- and 2-year relapse in the validation set, respectively.

Currently, lack of screening and early detection, the proneness for early relapse after radical resection and minimally effective systemic therapy remain major barriers to curing patients with PDAC [33]. Timely and accurate prediction of relapse even after operative intervention is difficult. Implementation of cutting-edge machine learning algorithms may help to identify at-risk patients, among whom more intensive surveillance, the use of adjuvant treatment, or even the inclusion of these patients into clinical trials may be considered. Nowadays, artificial intelligence (AI) research in healthcare is accelerating rapidly, with potential applications across almost every domain of medicine [34–36]. As an important branch of AI, machine learning allows computers to train models using large numbers of examples and may detect difficult-to-recognize patterns from complex dataset [37]. Unlike conventional regression-based approaches, machine learning algorithms are capable of capturing higher-order, non-linear inter-actions between predictors [38]. As a widely used model in biomedical analytics, SVM creates a set of hyperplanes for each feature in an infinite dimensional space, and fits linear or nonlinear models that most effectively discriminate between the values of a binary output variable [39]. Its effectiveness has been proved in studies to predict the recurrence of various diseases [40–42]. KNN is another stringent methodology for classification and regression. Reports have also demonstrated its promising role in prognostic research [43–45]. It can be useful to weight the contributions of the neighbours, so that the nearer neighbours contribute more to the average than the more distant ones [46]. Our study allowed for the comparison of multiple learning algorithms to identify the approach with the most favorable performance.

To the best of our knowledge, this is the first study to develop and compare machine learning-based models to predict relapse risk of pancreatic ductal adenocarcinoma after radical resection from multi-institutional datasets. Predictive nomograms based on conventional regression methods have been built for early recurrence after pancreatectomy in resectable pancreatic cancer [9, 12]. Kim et al. established a nomogram to predict the probability of recurrence within 12 months after surgery in single medical center with AUROC = 0.655 [9]. While in our study, we constructed and externally validated a predictive SVM model for 1-year relapse risk with AUROC = 0.733 and a KNN model for 2-year relapse risk with AUROC = 0.689 using stringent statistical method. Another work by Guo et al. redefined early recurrence as the first 162 days postoperatively on a basis of its own cohort, which made it difficult to compare results across studies and different institutions [12]. Particularly, it is understandable that this study did not include histopathologic data in its Cox proportional hazards regression model for the purpose of guiding preoperative decision-making concerning the use of neoadjuvant therapy. Other reports regarding this topic also have their own specific drawbacks with either a very small sample size of less than 40 [30] or lack of external validation [10]. In addition, recent research has revealed the links between radiomics and underlying tumor biology in PDAC, which are strongly correlated with tumor phenotype [47], response to treatment [48], and prognosis [49–51]. However, the steps of image texture analysis and manual contouring of region of interests (ROIs) are still tedious, laborious and time-consuming, which is inconvenient for clinical practice at present and has ample room to improve in the future.

Certain limitations of this study and the results need to be discussed. First, given the retrospective nature of our study, there might be some selection bias existing because of its inherent flaws. Second, despite the low incidence of PDAC, the relatively limited sample size included in the training and validation dataset might impair the accuracy for quantifying interpatient variability effects. Both two models showed high sensitivity with a trade-off that the specificity might be sacrificed in a certain level, which is relevant to the threshold selection when performing binary classification [52]. More larger and balanced cohorts will be collected from multiple medical centers in the future to further establish the robustness of the proposed models. Third, limitations in the interpretability of inner workings of models currently poses a severe bottleneck in implementing cutting-edge machine learning techniques in biomedical research [34, 53]. We need to keep pursuing a better understanding of the complex and evolving relationship between physicians and human-centred AI tools in the live clinical environment, thus providing better outcomes to our patients [54].

In conclusion, we employed machine learning algorithms to construct models integrating clinicopathological characteristics to predict the relapse risk of PDAC after radical resection. And we have externally validated the prediction capacity of our models in independent groups from other medical institutions. Machine learning systems can provide critical prognostic prediction for patients with PDAC after radical resection, and the use of predictive algorithms may offer promising clinical decision support for both practitioners and patients.

## Supplementary Information

**Additional file 1: Figure S1.** Comparisons of AUROC of different models to predict 1- and 2-year relapse in training set (a: 1-year relapse, b: 2-year relapse). **Figure S2.** Calibration curves of SVM model (A: training set; B: validation set) to predict 1-year relapse and KNN model (C: training set; D: validation set) to predict 2-year relapse. **Table S1.** The ranges of training parameters for grid search in different models. **Table S2.** Comparison of characteristics between patients with and without 1-year relapse in training set. **Table S3.** Comparison of characteristics between patients with and without 2-year relapse in training set. **Table S4.** The optimal parameters for different models to predict relapse risks of PDAC. **Table S5.** Performance of models built on all 32 variables in the validation set. **Table S6.** Performance of models built on variables from lasso analysis in the validation set.

## Data Availability

The datasets generated during and analysed during the current study are available in the Code Ocean (https://codeocean.com/capsule/2968380/tree).

## Abbreviations

PDAC: Pancreatic ductal adenocarcinoma
AI: Artificial intelligence
CT: Computed tomography
MRI: Magnetic resonance imaging
PET: Positron emission tomography
RFS: Relapse-free survival
OS: Overall survival
RF: Ramdom forest
SVM: Support vector machine
GBM: Gradient boosting machine
NN: Neural network
KNN: K neighbor algorithm
AUROC: Area under the receiver operating characteristic curve
PPV: Positive predictive value
NPV: Negative predictive value
RMSE: Root mean squared error
CI: Confidence interval
BMI: Body mass index
CEA: Carcinoembryonic antigen
CA: Cancer antigen
WBC: White blood cell
Hb: Hemoglobin
Plt: Platelet
Neut: Neutrophil
Lymph: Lymphocyte
Mono: Monocyte
Alb: Albumin
Glb: Globulin
AGR: Albumin-globulin ratio
NLR: Neutrophil–lymphocyte ratio
LMR: Lymphcyte-monocyte ratio
PLR: Platelet-lymphocyte ratio
AST: Aspartate transaminase
ALT: Alanine transaminase
ALP: Alkaline phosphatase
GGT: Gamma-glutamyltransferase
TB: Total bilirubin
DB: Direct bilirubin
VI: Vascular invasion
PI: Perineural invasion
ATI: Adipose tissue invasion
OS: Overall survival
RFS: Relapse-free survival
