# Supplementary material for: Multi-institutional development and external validation of machine learning-based models to predict relapse risk of pancreatic ductal adenocarcinoma after radical resection
Source: J Transl Med. 2021 Jun 30;19:281. doi: 10.1186/s12967-021-02955-7 (PMC8243478; doi:10.1186/s12967-021-02955-7)
Supplement: Supplementary file 1 — Additional file 1: Figure S1. Comparisons of AUROC of different models to predict 1- and 2-year relapse in training set (a: 1-year relapse, b: 2-year relapse). Figure S2. Calibration curves of SVM model (A: training set; B: validation set) to predict 1-year relapse and KNN model (C: training set; D: validation set) to predict 2-year relapse. Table S1. The ranges of training parameters for grid search in different models. Table S2. Comparison of characteristics between patients with and without 1-year relapse in training set. Table S3. Comparison of characteristics between patients with and without 2-year relapse in training set. Table S4. The optimal parameters for different models to predict relapse risks of PDAC. Table S5. Performance of models built on all 32 variables in the validation set. Table S6. Performance of models built on variables from lasso analysis in the validation set. [file 12967_2021_2955_MOESM1_ESM.docx]

**Additional Figure S1** Comparisons of AUROC of different models to predict 1- and 2-year relapse in training set (a: 1-year relapse, b: 2-year relapse)

**Additional Figure S2** Calibration curves of SVM model (A: training set; B: validation set) to predict 1-year relapse and KNN model (C: training set; D: validation set) to predict 2-year relapse

**
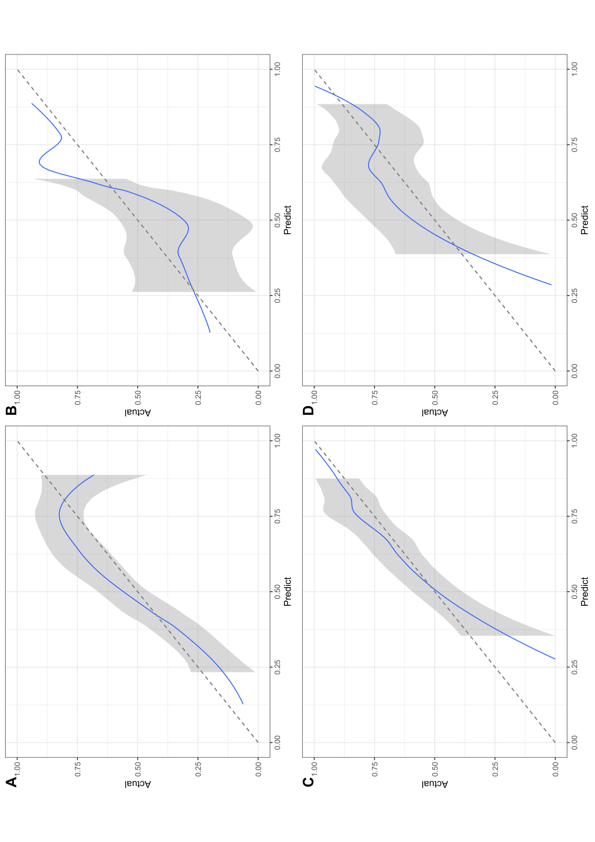
**

**Additional Table S1.** The ranges of training parameters for grid search in different models.

| Models | Parameters |
| --- | --- |
| RF | mtry=c(1:20)  ntree=c(10,20,30,40,50,60,70,80,90,100,200,300,400,500) |
| SVM | sigma= c(0.01,0.03,0.05,0.07,0.10,0.15)  C= c(0.1,0.3,0.5,0.7,0.9,1) |
| GBM | interaction.depth = c(1,3,5,7,9)  n.trees = (1:30)*30  shrinkage = c(0.05,0.1,0.2)  n.minobsinnode = c(10,20,30) |
| NNET | size=c(1:20)  decay=c(5e-04,0.1,0.3,0.5) |
| KNN | k = seq(1,100,by=2) |

**Additional Table S2.** Comparison of characteristics between patients with and without 1-year relapse in training set

| Variables |  | With (n=106) | Without (n=77) | *p* |
| --- | --- | --- | --- | --- |
| Age(years) | median (q1-q3) | 63.5 (56.0-70.0) | 63.0 (55.0-69.0) | 0.683 |
| Gender | male (%) | 68 (64.2) | 47 (61.0) | 0.783 |
|  | female (%) | 38 (35.8) | 30 (39.0) |  |
| BMI(kg/m^2^) | median (q1-q3) | 22.4 (20.2-23.9) | 22.1 (20.6-23.8) | 0.716 |
| CEA(ng/mL) | <5 (%) | 80 (75.5) | 60 (77.9) | 0.834 |
|  | ≥5 (%) | 26 (24.5) | 17 (22.1) |  |
| CA199(U/mL) | <37 (%) | 22 (20.8) | 27 (35.1) | 0.047 |
|  | ≥37 (%) | 84 (79.2) | 50 (64.9) |  |
| CA125(U/mL) | <35 (%) | 84 (79.2) | 63 (81.8) | 0.807 |
|  | ≥35 (%) | 22 (20.8) | 14 (18.2) |  |
| WBC(*10^9) | median (q1-q3) | 6.0 (4.9-7.1) | 5.8 (4.7-7.4) | 0.395 |
| Hb(g/L) | median (q1-q3) | 129.0 (117.3-141.8) | 124.0 (113.0-136.0) | 0.356 |
| Plt(*10^9) | median (q1-q3) | 187.0 (150.3-230.0) | 191.0 (158.0-234.0) | 0.505 |
| Neut(*10^9) | median (q1-q3) | 4.0 (3.0-4.8) | 3.7 (2.8-4.9) | 0.574 |
| Lymp(*10^9) | median (q1-q3) | 1.4 (1.0-1.7) | 1.4 (1.1-1.7) | 0.409 |
| Mono(*10^9) | median (q1-q3) | 0.5 (0.4-0.5) | 0.5 (0.4-0.6) | 0.268 |
| Alb(*10^9) | median (q1-q3) | 40.6 (37.8-43.4) | 38.9 (36.5-43.2) | 0.371 |
| Glb(*10^9) | median (q1-q3) | 27.4 (24.9-29.4) | 26.3 (24.1-30.8) | 0.904 |
| AGR | median (q1-q3) | 1.5 (1.4-1.7) | 1.5 (1.3-1.6) | 0.372 |
| NLR | median (q1-q3) | 2.9 (2.0-4.2) | 2.7 (1.9-4.2) | 0.872 |
| LMR | median (q1-q3) | 2.8 (2.1-4.0) | 3.1 (1.9-4.3) | 0.929 |
| PLR | median (q1-q3) | 134.0 (109.9-184.3) | 132.6 (106.3-183.5) | 0.824 |
| AST(U/L) | median (q1-q3) | 43.0 (21.0-124.8) | 45.0 (20.0-95.0) | 0.382 |
| ALT(U/L) | median (q1-q3) | 51.5 (17.0-202.5) | 47.0 (16.0-191.0) | 0.612 |
| ALP(U/L) | median (q1-q3) | 164.0 (87.5-387.0) | 136.0 (94.0-391.0) | 0.670 |
| GGT(U/L) | median (q1-q3) | 168.5 (25.0-678.5) | 115.0 (25.0-735.0) | 0.865 |
| TB(μmol/L) | median (q1-q3) | 22.6 (11.8-178.4) | 22.1 (13.1-174.4) | 0.707 |
| DB(μmol/L) | median (q1-q3) | 5.5 (2.6-107.4) | 6.8 (2.6-103.9) | 0.614 |
| Location | Head-Isthmus (%) | 85 (80.2) | 54 (70.1) | 0.162 |
|  | Body-Tail (%) | 21 (19.8) | 23 (29.9) |  |
| Margin | R0 (%) | 100 (94.3) | 76 (98.7) | 0.241 |
|  | R1 (%) | 6 (5.7) | 1 (1.3) |  |
| T stage | 1 (%) | 27 (25.5) | 16 (20.8) | 0.751 |
|  | 2 (%) | 49 (46.2) | 37 (48.1) |  |
|  | 3 (%) | 30 (28.3) | 24 (31.2) |  |
| N stage | 0 (%) | 47 (44.3) | 58 (75.3) | <0.001 |
|  | 1 (%) | 41 (38.7) | 15 (19.5) |  |
|  | 2 (%) | 18 (17.0) | 4 ( 5.2) |  |
| VI | yes (%) | 61 (57.5) | 22 (28.6) | <0.001 |
|  | no (%) | 45 (42.5) | 55 (71.4) |  |
| PI | yes (%) | 87 (82.1) | 56 (72.7) | 0.183 |
|  | no (%) | 19 (17.9) | 21 (27.3) |  |
| ATI | yes (%) | 55 (51.9) | 27 (35.1) | 0.035 |
|  | no (%) | 51 (48.1) | 50 (64.9) |  |
| Differentiation | well (%) | 13 (12.3) | 24 (31.2) | 0.005 |
|  | moderate (%) | 86 (81.1) | 47 (61.0) |  |
|  | poor or undifferentiated (%) | 7 ( 6.6) | 6 ( 7.8) |  |
| OS | median (q1-q3) | 12.0 (8.0-18.0) | 34.5 (25.8-48.3) | <0.001 |
| RFS | median (q1-q3) | 7.0 (4.0-10.0) | 28.0 (18.8-42.3) | <0.001 |

Abbreviation: BMI=body mass index; CEA=carcinoembryonic antigen; CA=cancer antigen; WBC=white blood cell; Hb=Hemoglobin; Plt=Platelet; Neut=neutrophil; Lymph=lymphocyte; Mono=monocyte; Alb=albumin; Glb=globulin; AGR=albumin-globulin ratio; NLR=neutrophil-lymphocyte ratio; LMR=lymphcyte-monocyte ratio; PLR = platelet-lymphocyte ratio; AST=aspartate transaminase; ALT=alanine transaminase ; ALP=alkaline phosphatase; GGT=[gamma-glutamyltransferase](file:////Users/XiaweiLi/Documents\x/dic://gamma-glutamyltransferase%20GGT); TB=total bilirubin; DB=direct bilirubin; VI=vascular invasion; PI=perineural invasion; ATI=adipose tissue invasion; OS=overall survival; RFS=relapse-free survival.

**Additional Table S3.** Comparison of characteristics between patients with and without 2-year relapse in training set

| Variables |  | With (n=138) | Without (n=45) | *p* |
| --- | --- | --- | --- | --- |
| Age(years) | median (q1-q3) | 63.0 (56.0-69.0) | 64.0 (56.0-73.0) | 0.772 |
| Gender | male (%) | 90 (65.2) | 25 (55.6) | 0.324 |
|  | female (%) | 48 (34.8) | 20 (44.4) |  |
| BMI(kg/m^2^) | median (q1-q3) | 22.2 (20.1-23.8) | 22.5 (20.8-24.5) | 0.221 |
| CEA(ng/mL) | <5 (%) | 103 (74.6) | 37 (82.2) | 0.401 |
|  | ≥5 (%) | 35 (25.4) | 8 (17.8) |  |
| CA199(U/mL) | <37 (%) | 31 (22.5) | 18 (40.0) | 0.035 |
|  | ≥37 (%) | 107 (77.5) | 27 (60.0) |  |
| CA125(U/mL) | <35 (%) | 113 (81.9) | 34 (75.6) | 0.477 |
|  | ≥35 (%) | 25 (18.1) | 11 (24.4) |  |
| WBC(*10^9) | median (q1-q3) | 5.8 (4.7-7.1) | 6.4 (5.2-7.6) | 0.548 |
| Hb(g/L) | median (q1-q3) | 128.5 (117.0-142.0) | 124.0 (112.0-134.0) | 0.085 |
| Plt(*10^9) | median (q1-q3) | 184.5 (147.3-227.5) | 197.0 (170.0-242.0) | 0.181 |
| Neut(*10^9) | median (q1-q3) | 3.9 (2.8-4.7) | 4.2 (3.3-5.1) | 0.177 |
| Lymp(*10^9) | median (q1-q3) | 1.4 (1.1-1.7) | 1.5 (1-1.7) | 0.435 |
| Mono(*10^9) | median (q1-q3) | 0.5 (0.4-0.5) | 0.5 (0.4-0.7) | 0.034 |
| Alb(*10^9) | median (q1-q3) | 40.5 (37.8-43.8) | 38.5 (35.3-42.2) | 0.015 |
| Glb(*10^9) | median (q1-q3) | 27.3 (24.6-29.5) | 26.3 (24.1-29.2) | 0.530 |
| AGR | median (q1-q3) | 1.5 (1.4-1.7) | 1.5 (1.3-1.6) | 0.046 |
| NLR | median (q1-q3) | 2.8 (1.9-4.0) | 2.7 (2.1-4.9) | 0.425 |
| LMR | median (q1-q3) | 3.0 (2.1-4.2) | 2.9 (1.8-3.6) | 0.227 |
| PLR | median (q1-q3) | 137.1 (104.9-183.0) | 135.5 (108.2-201.5) | 0.805 |
| AST(U/L) | median (q1-q3) | 44.0 (20.0-117.8) | 46.0 (21.0-92.0) | 0.288 |
| ALT(U/L) | median (q1-q3) | 51.5 (17.0-202.5) | 47.0 (18.0-156.0) | 0.406 |
| ALP(U/L) | median (q1-q3) | 158.0 (89.3-386.3) | 129.0 (91.0-404.0) | 0.652 |
| GGT(U/L) | median (q1-q3) | 162.5 (25.0-743.5) | 124.0 (25.0-594.0) | 0.639 |
| TB(μmol/L) | median (q1-q3) | 18.6 (11.8-173.1) | 28.2 (14.1-180.8) | 0.716 |
| DB(μmol/L) | median (q1-q3) | 5.3 (2.5-103.5) | 8.8 (2.9-106.8) | 0.812 |
| Location | Head-Isthmus (%) | 107 (77.5) | 32 (71.1) | 0.500 |
|  | Body-Tail (%) | 31 (22.5) | 13 (28.9) |  |
| Margin | R0 (%) | 132 (95.7) | 44 (97.8) | 1.000 |
|  | R1 (%) | 6 (4.3) | 1 (2.2) |  |
| T stage | 1 (%) | 32 (23.2) | 11 (24.4) | 0.924 |
|  | 2 (%) | 66 (47.8) | 20 (44.4) |  |
|  | 3 (%) | 40 (29.0) | 14 (31.1) |  |
| N stage | 0 (%) | 66 (47.8) | 39 (86.7) | <0.001 |
|  | 1 (%) | 50 (36.2) | 6 (13.3) |  |
|  | 2 (%) | 22 (15.9) | 0 ( 0.0) |  |
| VI | yes (%) | 75 (54.3) | 8 (17.8) | <0.001 |
|  | no (%) | 63 (45.7) | 37 (82.2) |  |
| PI | yes (%) | 109 (79.0) | 34 (75.6) | 0.783 |
|  | no (%) | 29 (21.0) | 11 (24.4) |  |
| ATI | yes (%) | 68 (49.3) | 14 (31.1) | 0.051 |
|  | no (%) | 70 (50.7) | 31 (68.9) |  |
| Differentiation | well (%) | 25 (18.1) | 12 (26.7) | 0.158 |
|  | moderate (%) | 105 (76.1) | 28 (62.2) |  |
|  | poor or undifferentiated (%) | 8 ( 5.8) | 5 (11.1) |  |
| OS | median (q1-q3) | 14.5 (9.0-23.0) | 41.0 (34.8-60.3) | <0.001 |
| RFS | median (q1-q3) | 8.0 (4.3-12.0) | 38.0 (30.0-58.5) | <0.001 |

Abbreviation: BMI=body mass index; CEA=carcinoembryonic antigen; CA=cancer antigen; WBC=white blood cell; Hb=Hemoglobin; Plt=Platelet; Neut=neutrophil; Lymph=lymphocyte; Mono=monocyte; Alb=albumin; Glb=globulin; AGR=albumin-globulin ratio; NLR=neutrophil-lymphocyte ratio; LMR=lymphcyte-monocyte ratio; PLR = platelet-lymphocyte ratio; AST=aspartate transaminase; ALT=alanine transaminase ; ALP=alkaline phosphatase; GGT=[gamma-glutamyltransferase](file:////Users/XiaweiLi/Documents\x/dic://gamma-glutamyltransferase%20GGT); TB=total bilirubin; DB=direct bilirubin; VI=vascular invasion; PI=perineural invasion; ATI=adipose tissue invasion; OS=overall survival; RFS=relapse-free survival.

**Additional Table S4.** The optimal parameters for different models to predict relapse risks of PDAC

|  | **1-year relapse** | **2-year relapse** |
| --- | --- | --- |
| **RF** | mtry=3  ntree = 60 | mtry=2  ntree = 80 |
| **SVM** | sigma = 0.01  C = 0.9 | sigma = 0.03  C = 0.5 |
| **GBM** | n.trees = 810  interaction.depth = 7  shrinkage = 0.2  n.minobsinnode = 20 | n.trees = 120  interaction.depth = 5  shrinkage = 0.05  n.minobsinnode = 20 |
| **NNET** | size = 3  decay = 0.5 | size = 3  decay = 0.5 |
| **KNN** | k = 11 | k = 21 |

**Additional Table S5.** Performance of models built on all 32 variables in the validation set

| **1-year relapse** | | |  |  |  |  |  |  |  |  |
| --- | --- | --- | --- | --- | --- | --- | --- | --- | --- | --- |
| Method | AUC | AUClower | AUCupper | Sensitivity | Specificity | Accuracy | PPV | NPV | F1 | RMSE |
| LR | 0.617 | 0.493 | 0.745 | 0.755 | 0.500 | 0.658 | 0.712 | 0.556 | 0.733 | 0.517 |
| RF | 0.626 | 0.497 | 0.748 | 0.612 | 0.500 | 0.570 | 0.667 | 0.441 | 0.638 | 0.477 |
| SVM | 0.666 | 0.536 | 0.784 | 0.837 | 0.400 | 0.671 | 0.695 | 0.600 | 0.759 | 0.472 |
| GBM | 0.602 | 0.471 | 0.724 | 0.776 | 0.267 | 0.582 | 0.633 | 0.421 | 0.697 | 0.478 |
| NN | 0.681 | 0.559 | 0.801 | 0.837 | 0.433 | 0.684 | 0.707 | 0.619 | 0.766 | 0.487 |
| KNN | 0.677 | 0.559 | 0.795 | 0.776 | 0.433 | 0.646 | 0.691 | 0.542 | 0.731 | 0.464 |
| **2-year relapse** | | |  |  |  |  |  |  |  |  |
| Method | AUC | AUClower | AUCupper | Sensitivity | Specificity | Accuracy | PPV | NPV | F1 | RMSE |
| LR | 0.622 | 0.472 | 0.769 | 0.864 | 0.300 | 0.722 | 0.785 | 0.429 | 0.823 | 0.488 |
| RF | 0.503 | 0.371 | 0.635 | 0.966 | 0.000 | 0.722 | 0.740 | 0.000 | 0.838 | 0.447 |
| SVM | 0.590 | 0.439 | 0.739 | 0.949 | 0.100 | 0.734 | 0.757 | 0.400 | 0.842 | 0.445 |
| GBM | 0.670 | 0.541 | 0.788 | 0.932 | 0.150 | 0.734 | 0.764 | 0.429 | 0.840 | 0.429 |
| NN | 0.614 | 0.465 | 0.759 | 0.915 | 0.250 | 0.747 | 0.783 | 0.500 | 0.844 | 0.465 |
| KNN | 0.673 | 0.530 | 0.804 | 1.000 | 0.000 | 0.747 | 0.747 | NA | 0.855 | 0.428 |

**Additional Table S6.** Performance of models built on variables from lasso analysis in the validation set

| **1-year relapse** | | |  |  |  |  |  |  |  |  |
| --- | --- | --- | --- | --- | --- | --- | --- | --- | --- | --- |
| Method | AUC | AUClower | AUCupper | Sensitivity | Specificity | Accuracy | PPV | NPV | F1 | RMSE |
| LASSO-LR | 0.742 | 0.627 | 0.849 | 0.776 | 0.567 | 0.696 | 0.745 | 0.607 | 0.760 | 0.470 |
| RF | 0.663 | 0.535 | 0.779 | 0.837 | 0.367 | 0.658 | 0.683 | 0.579 | 0.752 | 0.537 |
| SVM | 0.735 | 0.611 | 0.836 | 0.878 | 0.367 | 0.684 | 0.694 | 0.647 | 0.775 | 0.450 |
| GBM | 0.715 | 0.594 | 0.821 | 0.857 | 0.367 | 0.671 | 0.689 | 0.611 | 0.764 | 0.450 |
| NN | 0.695 | 0.577 | 0.819 | 0.878 | 0.367 | 0.684 | 0.694 | 0.647 | 0.775 | 0.454 |
| KNN | 0.579 | 0.440 | 0.714 | 0.796 | 0.367 | 0.633 | 0.672 | 0.524 | 0.729 | 0.505 |
| **2-year relapse** | | |  |  |  |  |  |  |  |  |
| Method | AUC | AUClower | AUCupper | Sensitivity | Specificity | Accuracy | PPV | NPV | F1 | RMSE |
| LASSO-LR | 0.636 | 0.487 | 0.776 | 0.915 | 0.200 | 0.734 | 0.771 | 0.444 | 0.837 | 0.449 |
| RF | 0.649 | 0.513 | 0.782 | 0.898 | 0.150 | 0.709 | 0.757 | 0.333 | 0.822 | 0.431 |
| SVM | 0.563 | 0.404 | 0.717 | 0.898 | 0.200 | 0.722 | 0.768 | 0.400 | 0.828 | 0.471 |
| GBM | 0.659 | 0.521 | 0.778 | 0.864 | 0.150 | 0.684 | 0.750 | 0.273 | 0.803 | 0.444 |
| NN | 0.595 | 0.445 | 0.736 | 0.847 | 0.300 | 0.709 | 0.781 | 0.400 | 0.813 | 0.475 |
| KNN | 0.653 | 0.502 | 0.806 | 0.932 | 0.150 | 0.734 | 0.764 | 0.429 | 0.840 | 0.425 |
